# Vulnerability of rural households to climate-induced shocks in Lokka Abaya district, Sidama zone, southern Ethiopia

**DOI:** 10.4102/jamba.v13i1.1051

**Published:** 2021-05-25

**Authors:** Muluken Mekuyie

**Affiliations:** 1 Department of General Forestry, Wondo Genet College of Forestry and Natural Resources, Hawassa University, Hawassa, Ethiopia

**Keywords:** adaptive capacity, vulnerability index, exposure, drought, vulnerable households, sensitivity, rural community

## Abstract

This study was conducted in rural communities of Lokka Abaya district, Sidama zone, southern Ethiopia to assess vulnerability status of men and women households to climate-induced shocks and stress. This article is based on household survey, focus group discussion and key informant interviews. A total of 258 smallholder farmers were selected from three villages using stratified random sampling. A combination of social, economic and environmental indicators was employed to develop the vulnerability index of each household head and estimate quantitatively that is vulnerability is estimated as a function of adaptive capacity, exposure and sensitivity of households. The results indicated that farmers had poor access to public services including access to affordable credit, market, health services and climate information. The survey revealed that droughts, floods, soil erosion, pests and diseases were climate-related challenges in the study area. Regarding vulnerability of households to climate variability, results indicated that around 8.5% and 18.2% of male- and female-headed households, respectively, were highly vulnerable whilst 41% and 45.5% of male- and female-headed households, respectively, were moderately vulnerable. The results confirmed that 37.7% and 27.3% of male- and female-headed households, respectively, were less vulnerable. The rest 12.8% men and 9% women were not vulnerable. Therefore, there is a need to enhance access to affordable credit, market, climate information, health, income diversification of farmers, soil and water conservation and afforestation of hilly areas if farmers need to be climate resilient.

## Introduction

There is clear scientific evidence that the earth’s climate is changing (Intergovernmental Panel in Climate Change [IPCC] 2007; Spore 2008). Climate-induced shocks and stresses are amongst the greatest developmental challenges with multiple impacts on basic human support systems such as agriculture, forest and water resources (Amsalu & Adem 2009).

Over the years, the frequency of the climate change and variation in terms of temperature and rainfall has been increasing (Chibinga et al. 2012). Such changes would alter the natural climate and environmental systems, leading to increased frequency of extreme weather events such as droughts, storms and flooding (Zhai & Zhuang 2009).

Despite worldwide coverage of climate change impact, it is expected to have serious environmental, economic and social impacts particularly on rural communities in Africa whose livelihoods depend on the use of natural resources (Gbetibouo 2009; Thornton et al. 2008). Africa as a whole is considered to be amongst the most vulnerable regions to climate variability and change. This has been attributed to the continent’s low adaptive capacity, lack of financial, institutional and technological capacity, overdependence on rainfed agriculture and existence of many other stressors (Collier, Conway & Venables 2008). Ethiopia is not exceptional regarding its vulnerability to climate change and variability.

Most policies targeted to develop and strengthen the adaptive capacity of communities. However, they have not recognised how male- and female-headed households respond to climate change (Alston 2013; Terry 2009), therefore, decision makers wrongly frame women and men issues in policy development (Arora-Jonsson 2014; Naess 2013). Differences in vulnerability and exposure arising from non-climatic factors such as geophysical, agro-ecological and socioeconomic factors shape differential risks to climate change (IPCC 2014).

To identify the most vulnerable gender group in society, access to resource and public services is crucial. Studies show that vulnerability to climate change and its impacts on communities are determined by whether the household is female-headed or male-headed, education level of households, access to credits and climate information (Moosa & Tuana 2014; Morchain et al. 2015; Rao 2016).

Therefore, building up an understanding of men and women vulnerability as rising up out of poverty and social discrimination, and socio-cultural practices in various political, geographical and historical settings, aside from climatic change and variability (Blaikie et al. 1994; Few 2007) is vital to understanding capacities of communities to cope with and adapt to climate change. On the other hand, a study on the various adaptive strategies employed by men and women households to secure their livelihoods, both in the short- and long-term (Shipton 1990), is however, still lacking. Access to assets is significant, but how these relate to women and men households in various situations needs investigation (Moosa & Tuana 2014).

This study was conducted in Lokka Abaya district where enset-coffee-based home gardens were the prevalent land use system combined with crop and livestock activities. However, recently there is a rapid shift away from the traditional enset-coffee-based home gardens to cash crop khat-based systems, particularly in densely populated areas of the Sidama zone (Mellise et al. 2018). The cash crop monoculture could increase the vulnerability of households to climate-related shocks and stresses. In addition, demographic pressure in Lokka Abaya district puts pressure on the land use system leading to reduction in available farmlands and declining soil fertility and may further worsen the effect of climate change on local communities by increasing their vulnerability to climate-related shocks.

Most studies in Ethiopia have investigated sector vulnerability to climate change and variability with little emphasis on vulnerability analysis of male- and female-headed households and their adaptation strategies in relation to climate change. Therefore, the present study aims to examine the vulnerability of male- and female-headed households to climate-induced shocks in Lokka Abaya district, Southern Nations, Nationalities and Peoples Regional State (SNNPRS) of Ethiopia. Results of the present study will offer specific gender vulnerability levels to climate-related shocks and stress. This, in turn, will be significant in developing strategies to address the specific needs of gender groups in decreasing vulnerability to climate-related risks. The study will also provide an input to local decision makers and development partners to bring women into the planning, financing and implementation of climate responses, involving adaptation and mitigation strategies.

The following section involves materials and methods of this study. It includes description of the study area, sampling techniques, data collection methods, approaches to analyse vulnerability of household heads to climate-related shocks and data analysis.

## Materials and methods

### Description of study area

**FIGURE 1:**
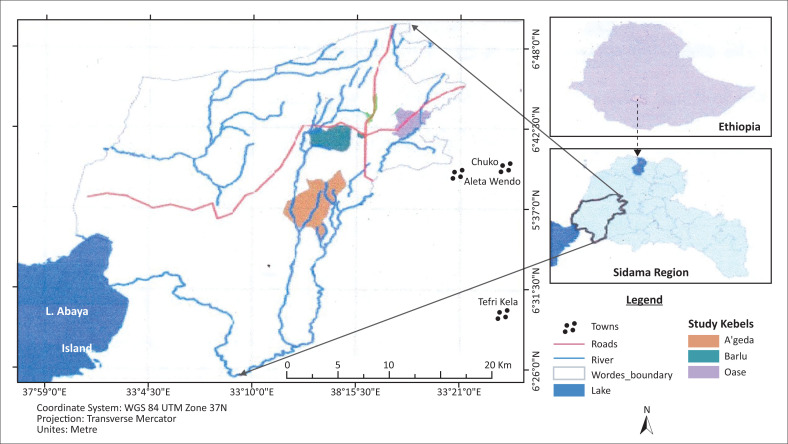
Map of the study area.

The study was conducted in Lokka Abaya, western border of the Sidama zone of the SNNPRS of Ethiopia, located at 6°17′25″ N latitude and 37°49′44″ E longitude (Figure 1). It is situated at 325 km southwest of the capital, Addis Ababa and 50 km southwest of regional city, Hawassa. The study area is characterised by bi-modal type of rainfall in which the short rainy season occurs from March to May, whilst the main rainy season occurs from June to September. Mean annual rainfall varies from 700 mm to 1877 mm and the mean annual temperature ranges from 26°C to 35°C. The area is also characterised by erratic rainfall, moisture stress and high temperature during the dry season (Central Statistical Agency [CSA] 2007). The altitude ranges from 1500 m to 1768 m above sea level. Agriculture is the principal source of livelihood for most of the population in the district. The soil type is mainly grey sandy loam and it is susceptible to erosion (United States Agency for International Development [USAID] 2005).

### Research design

This study area, Lokka Abaya district was purposely selected because high climate variability was reported by previous researchers in the area (Lalego, Ayalew & Kaske 2019). A stratified random sampling method was employed to select sampling villages. Stratification of villages was based on agro-ecology of the study area (lowland, midland and highland) whilst households were stratified based on gender (female- and male-headed households), and wealth status. Household heads were stratified into poor, medium and rich. The local people had some criteria for wealth category including farm size of a household head, livestock owned, income level, quality of the house and position of a household head in the community. For example, a household head was assumed to be poor if he or she had a farm size of less than 1 ha, owned not more than 1 Tropical Livestock Unit (TLU) livestock, poor quality of house and had no position in a community. From each agro-ecology, one village was randomly selected. Then, from each stratum, sample households were selected randomly. The list of female- and male-headed households was obtained from village level administrative offices. The study applied a simplified formula provided by Yamane (1967) to determine the required sample size at 95% confidence level, degree of variability = 0.5 and level of precision = 5%:

$$n=\frac{N}{1+N{\left(e\right)}^{2}}$$ [Eqn 1]

Where *n* is the total sample size, *N* is the population size (total household heads size) and e is the level of precision.

Then, depending on the total sample size (*n*) and total population size (total household heads size) (*N*), we calculated the sample size of household heads (*n*1) proportionally for each study site or village using the following formula:

$$n_{1}=\frac{N1(n)}{\Sigma N}$$ [Eqn 2]

where: *n*_1_ = distribution of sample size of household heads of each village, *N*1 = total size of household heads in each village, *n* = total sample size of this study (258) and Σ*N* = total household heads size of the study sites (three villages).

A structured questionnaire was administered face to face with selected female- and male-headed households. The collected data were coded and thereafter analysed using Statistical Packages for the Social Sciences (SPSS) (version 20). To complement the household questionnaire data, 15 individuals from selected villages and experts from the local Agriculture and Rural Development office and Disaster Prevention and Food Security Coordination Office were interviewed as key informants. Four focus group discussions were conducted separately with gender equality (four men and four women) from selected villages.

### Data collection

A pilot test run was carried out with local enumerators and key informants before the start of the household questionnaire survey, and the final questionnaires were revised and rephrased accordingly. The questionnaire used for the pre-test was omitted from the final data entry and analysis. The piloting was conducted to check the appropriateness of the tools and also whether the enumerators could manage the household survey without difficulty. Information on different characteristics was collected via interviewing of the sample female- and male-headed households. The survey involved information about household characteristics, household access to basic services, livelihood assets, income per household, climate change information, perception of local communities about climate-induced shocks and farm labour. To avoid misunderstanding, the household questionnaire survey was conducted in the local language by the enumerators.

### Data analysis

The completed interview schedule was coded and similarities as well as differences in the responses were viewed and noted. The SPSS was employed to analyse the quantitative data. Descriptive and inferential statistics were used in this study. With regard to descriptive statistics, frequencies and percentages were used in presenting information on household socio-economic characteristics. Chi-square tests were carried out to understand association between some qualitative variables. We used Principal Component analysis (PCA) to assign weights for selected indicators.

The present study employed vulnerability indicators to assess vulnerability of female- and male-headed households to climate-related shocks and stresses. Firstly, values of vulnerability indicators were normalised to make the indicator’s value within a similar range following the equation adopted by Gbetibouo & Ringler (2010); Nelson et al. (2010); Vincent (2004):

$$Normalised\;Value=\frac{Observed\;value-Mean}{Standard\;deviation}$$ [Eqn 3]

Then weights were assigned to the selected vulnerability indicators using PCA (Filmer & Pritchett 2001):

$$I_{j}=\sum_{i=1}^{K}b_{i}\left(\frac{a_{ji}-X_{i}}{S_{i}}\right)$$ [Eqn 4]

Where:

I = the index value

*b* = the weights from PCA

*a* = the individual value of the indicator

*x* = the mean value of the indicator

*s* = the standard deviation of the indicators.

Lastly, the vulnerability index of each household was calculated using the following equation (following IPCC 2012):

V=AC−(E+S) [Eqn 5]

Where:

V = the vulnerability index of each household

AC = the adaptive capacity index

E = the exposure index

S = the sensitivity index for the corresponding household.

## Results and Discussions

### Socio-economic characteristics of households

This study involved a total of 258 households of which 83% and 17% were male- and female-headed households, respectively. All household heads were in the range of 28–64 years old, indicating that they were in the productive age category. This result is in agreement with Seyoum (2015) in his study in three districts of the Sidama zone, southern Ethiopia who reported that the age of households was in the productive age category. The findings also showed that the average size of the family was 7.2 and 7.5 with a dependency ratio of 4 and 5 for male- and female-headed households, respectively. This was generally higher than the national average family size. Similar results were reported by Fenta, Jordaan & Melka (2019) in their study in the southern Afar region, Ethiopia. The results indicated that productive age category of the family members of male-headed households was better than female-headed households. In other words, there were sufficient working family members to support the dependent members in male-headed households compared to female-headed households. This might increase the vulnerability of women to climate-related risks compared to their counterparts. Concerning the education level of respondents, 10.88% and 36.36% of the male- and female-headed households, respectively, were illiterate. Furthermore, 28.57% and 18.18% of male- and female-headed households can read and write whilst 47.62% and 36.36% of men and women, respectively, attended primary first cycle (grade 1–4). The rest 12.93% and 9.1% of men and women, respectively, had joined primary second cycle (grade 5–8). This indicates that relatively female-headed households had poor access to education compared to men who could increase their vulnerability to climate change and variability. Studies indicated that illiterate people are more likely dependent on climate sensitive livelihoods and reluctant to adopt climate change adaptation measures (Fenta et al. 2019; Haile, Alemu & Kundhlande 2005).

The present study also assessed gender-based household’s access to public services including access to extension service, credit, health, market and climate information. Chi-square tests were carried out to understand the association between gender and public services. Results revealed that access to credit and climate information had significant association with gender at 0.05 significant level (Table 1). Findings showed that majority of male-headed households (89.8%) and female-headed households (81.8%) had access to extension services which implies that district experts and development agents (DAs) reached most of the households in the study area. The DAs assisted farmers by offering trainings on farmland preparation, soil and water conservation, crop cultivation and livestock production. According to the survey results, the DAs select those households with better education level and willing to accept agricultural technologies and train them on cultivation and harvesting of improved and local crops and production and management of improved livestock breeds, such as improved dairy cows, to enhance agricultural productivity. Then, the experts scale up these technologies using those successful model farmers and other demonstration sites through training, field visits and farmer to farmer experience sharing. Similar results were reported by Seyoum (2015) in his study in the Sidama zone, southern Ethiopia.

**TABLE 1:** Household’s access to public service by gender (%) and chi-square tests to test the relationship between gender and public services.

Variables	Response				χ^2^-test
	Male		Female		
	Yes	No	Yes	No	
Access to extension	89.8	10.2	81.8	18.2	0.085^ns^
Access to credit	40.8	59.2	9.1	90.9	0.007**
Access to health	69.2	30.8	65.7	34.3	0.376^ns^
Access to market	52.5	47.5	54.5	45.5	0.067^ns^
Access to climate information	49.0	51.0	18.2	81.8	0.013*

ns, non-significant.

* , Significance at α = 0.05;

** , significance at α = 0.01.

Results revealed that there was low access to credit for both men (40.8%) and women (9.1%) and especially credit access of women was very low which might reduce their adaptive capacity. Similar findings were reported by Carney (1999) and Seyoum (2015) who indicated that less access to social services such as credit, health and market in their studies was the most important barrier of adaptation strategies. Furthermore, regarding access to health, around 69.2% and 65.7% male-headed and female-headed households, respectively, had access to health centres in their nearby villages at a distance of 5 km – 10 km. In order to accomplish the day-to-day livelihood activities, the health status of a household is a significant component of human capital. It is obvious that if the family member of a household is sick, he or she could not carry out his or her livelihood activities besides the direct impacts on the well-being of a household and the expenses incurred for medications and treatments. Hence, this might increase vulnerability of households to climate-related risks. The results indicated that 83.9% and 71.8% of male- and female-headed households, respectively, encountered sickness in at least one member of their family for the last 1 year. The most common diseases complained by the communities were malaria and diarrhea. On an average, a household incurred a cost of 600.70 Ethiopian birr (ETB) for the last 1 year for medications and treatments. This result was in line with the findings of Seyoum (2015) who reported that most farmers in his study area, Sidama zone, southern Ethiopia were frequently affected by malaria.

Climate-related early warning information of a community is paramount to enhance its climate resilience.

However, in the present study, the findings revealed that household’s access to climate information was low. Only 49% and 18.2% of men and women had access to climate information through local DAs and radios which implies increased vulnerability of farmers to climate-related risks. In particular, female-headed households had very low access to climate information. Similarly, 52.5% men and 54.5% women had access to market in their nearby villages at a distance of 3 km to 10 km. The results are similar to the findings reported by Fenta et al. (2019) in their study in the Afar region, Ethiopia. The respondents complained that farmers in the study area were not getting their products price they expected because of the lack of market linkage and centre in their nearby village. In the study area, the livelihood of households is more dependent on cash crop production such as coffee and khat and fruits such as banana, avocado, mango and papaya than production of cereal crops. Most farmers sold cash crops and fruits and purchased their food crops from the nearby market. Hence, climate resilience and food security of households in the study area are closely linked with an access to market in the nearby village to sell their commodities at reasonable price and purchase their food crops. The results are in agreement with the study of Fenta, Jordaan and Melka (2018) in the southern Afar region who reported that access to basic services in this region was low. The results revealed that average farming experience of households was 24 and 20 years for male- and female-headed households, respectively.

Regarding assets of households, the average land holding of men and women households was 0.95 ha and 0.61 ha, respectively. Based on wealth category of households, the average farm size of households was 0.53 ha, 1.47 ha and 2.23 ha for the poor, medium and rich households, respectively (Table 2). The results revealed that 72.7% and 27.3% women were under poor and medium wealth category, respectively, whilst 77%, 49% and 21% men were under poor, medium and rich wealth category, respectively. Hence, 2.23 ha of land holding was owned by rich male-headed households which was above the national average of 1.18 ha whilst the average land holding of poor and medium households was below the national average. The chi-square test revealed that significant association was observed between wealth category and farm size at 0.05 significant level.

**TABLE 2:** Farm size of households by wealth category (ha).

Wealth	Minimum	Maximum	Mean ± SD
Poor	0.25	0.8	0.53 ± 0.25
Medium	0.30	1.87	1.47 ± 0.83
Rich	1.96	3.47	2.23 ± 1.2

SD, standard deviation.

On the other hand, the average livestock holding of households was 2.2 TLU and 1.9 TLU for male- and female-headed households, respectively. Livestock were an important source of cash for the smallholder farmers in the study area. For example, on an average, women and men earned 2173.91 ETB and 3755.25 ETB, respectively, from the sale of livestock and livestock products for the past 1 year.

Livestock were also an important source of protein and nutrient-rich products to complement the protein poor enset diet (*Enset ventricosum (Welw.) Cheesman*) and are the known staple food crops in the study area. Besides their role for ploughing, livestock played a critical role in providing organic fertilisers for crop production.

The average annual total income of men and women was 36137.20 ETB and 21389.55 ETB, respectively. Based on wealth category, the poor, medium and rich households earned an average total annual income of 13116.54 ETB, 36087.29 ETB and 119518.46 ETB, respectively (Table 3). The Least Significance Difference Test (LSD) showed that significance differences were observed amongst the mean income of poor, medium and rich households (Table 4). Majority of households (90%) earned their income from selling of cash crops such as khat (*Catha edulis* Forsk), coffee and fruits such as banana, mango and avocado. However, around 60% of households had more expenditure than their income because of the rising price of food crops such as maize (Zea mays) and Teff (Eragrostis tef) as most of their income was allocated to purchase food crops for their family. Hence, they were dependent on safety nets because of food shortage they faced. Tiwari (2013) also reported similar results in his study in rural areas of the Sidama zone, southern Ethiopia who indicated that the number of households involved in safety nets was increasing.

**TABLE 3:** Income of households by wealth.

Wealth	%	Minimum	Maximum	Mean ± SD
Poor	67.1	2846.00	24888.00	13116.54 ± 5306.97
Medium	17.7	23374.00	49571.00	36087.29 ± 7414.5
Rich	15.2	51321.00	218957.00	119518.46 ± 5022.22

SD, standard deviation.

**TABLE 4:** Mean income comparisons using the Least Significance Difference Test amongst wealth category of household heads.

(I) Wealth category of the HH	(J) Wealth category of the HH	Mean difference (I−J)	Sig.
Poor	Medium	−22970.75*	0.006
	Rich	−106401.92*	0.000
Medium	Poor	22970.75*	0.006
	Rich	−83431.17*	0.000
Rich	Poor	106401.92*	0.000
	Medium	83431.17*	0.000

HH, household head; Sig., significance.

* , The mean difference is significance at the 0.01 level.

### Perceived climate-induced shocks in the study area

The local people reported three main climate-induced shocks in the study area, adversely impacting their livelihood. These were drought, flood, soil degradation, crop pests and diseases. The survey results indicated that 81.8% of the households had been experiencing drought four times for the past 10 years. The local smallholder farmers complained that the drought occurred frequently. The farmers further complained that the onset and cessation of rainfall became irregular during the normal seasons when there was no drought. Sometimes, the rain comes early before the beginning of the normal season and sometimes, it comes very late and ceased within short period of time. Seyoum (2015) also reported similar results in his study in neighbouring districts of the present study area in the Sidama zone, southern Ethiopia which indicated that more than 80% rural households recognised increased occurrence of drought and late onset of rainfall. Besides, 58% household’s farmlands had exposure to soil degradation because of climate-induced erosion aggravated by other non-climatic factors such as erosion-induced traditional farming practices. The results are supported by the findings of Osore and Moges (2014) in their study in Dale district, Sidama zone, southern Ethiopia who reported that heavy rains cleared top soils of farmlands decreasing crop productivity, caused the formation of gullies, dissection of farms and deposition of sediments on growing crops. Around 30% of households were affected by flooding two to three times for the past 10 years which caused loss of crops and livestock. Another emerging climate-related risk perceived by the local people was crop pests and diseases which affected 42% of households in the study area.

### Components of vulnerability

The vulnerability of households was determined using PCA based on three components including adaptive capacity, exposure and sensitivity following the IPCC (2012) equation. Principal Component analysis was conducted after evaluating the suitability of the data for PCA based on the Kaiser-Meyer-Olkin (KMO) and Bartt’s tests values. Li and Weng (2007) suggested that it is appropriate to perform PCA if the Bartlett’s value is less than one and the KMO value is greater than 0.5. In this study, the KMO value was 0.68, showing the acceptance of the model and the Bartlett’s test was significant (*p* < 0.01), indicating the suitability of factor analysis for the data.

#### Indicators of adaptive capacity

**FIGURE 2:**
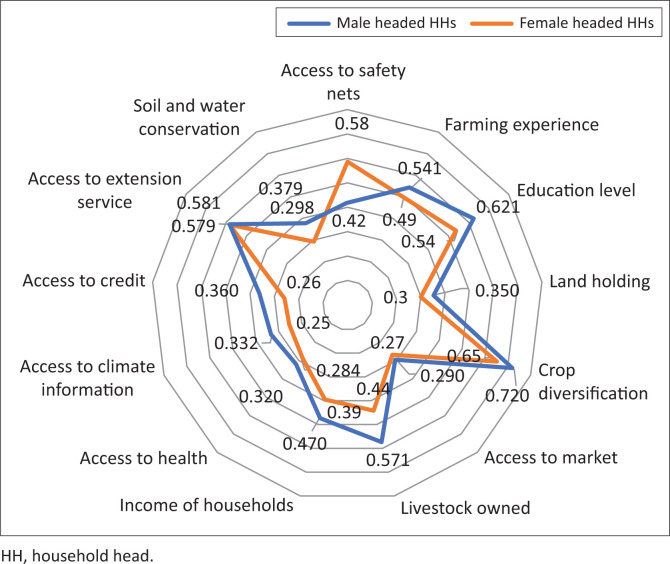
Factor loadings of observed variables of adaptive capacity of households.

Thirteen observed variables were included to develop the index of adaptive capacity of households as presented (Figure 2). Results showed how each observed variable contributed to the adaptive capacity of men and women to adapt and cope with climate-related hazards.

As indicated, crop diversification with values of 0.72 and 0.65 for men and women, respectively, (Figure 2) has the largest contribution in enhancing adaptive capacity of both male- and female-headed households and hence, reducing their vulnerability to climate-related risks such as drought. In the present study area, it is common to find a food crop, enset (*Enset ventricosum (Welw.) Cheesman*) grows together with cash crops such as coffee and Khat in farmlands of farmers. It was observed that on an average, 10.3% and 63.6% of men and women, respectively, grew three crops whilst 26.5% and 36.4% of men and women grew four crops. The rest 63.2% of men grew ≥ five crops.

This implies that male-headed households participated more than female-headed households in crop diversification and hence, men were with more capacity to reduce their vulnerability to climate-related hazards.

The other variables with the second largest factor loadings were education level of households and access to safety nets (which is of course short-term coping mechanism) for male- and female-headed households, respectively. The larger factor loading of access to safety nets for women implies their high dependency on aid and high vulnerability to climate-related hazards although the aid assists them to cope with short-term shocks. On the other hand, literacy level of men was relatively high and has larger contribution to enhance the adaptive capacity of men. However, the factor loading of education level of women was relatively low. This is explained by the fact that 36.36% of female-headed households were illiterate. This reveals that female-headed households were relatively with lower adaptive capacity than their counterparts. Studies indicated that those households with lower literacy had lower ability to adopt improved technologies (Fenta et al. 2019; Gebrehiwot & Van der Veen 2013; McCarthy et al. 2001).

The results indicated that access to extension service, livestock owned and farming experience and income level of households were also other variables with moderate factor loadings to enhance adaptive capacity of men and women. The other indicators with least factor loadings were soil and water conservation activities, access to credit and market, farm size, access to climate information and access to health services which contributed less to the adaptive capacity of men and women in the study area. As discussed above (Table 1), access to public services including access to credit and market, health and climate information was very low, implying their low contribution to enhance adaptive capacity of farmers in the study area. Shortage of farmland was another problem because of high population pressure in the area and hence, its contribution to enhance the adaptive capacity of men and women was relatively low.

#### Indicators of sensitivity and exposure

**FIGURE 3:**
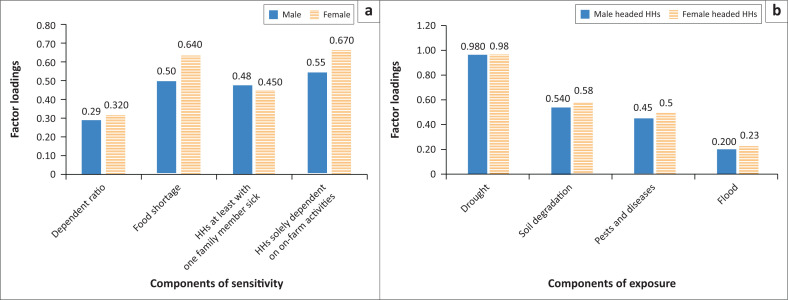
Factor loadings of indicators of sensitivity and exposure households.

In this study, we analysed four indicators of sensitivity that increase vulnerability of smallholder farmers and four climate-related hazards whereby households had been exposed. The factor loadings of these observed variables of sensitivity and exposure are presented (Figure 3).

The results indicated that female-headed households were more sensitive than their counterparts (Figure 3) as the factor loadings of all indicators of sensitivity in the case of women were larger than men implying their increased vulnerability to climate-induced risks. Households were solely dependent on agriculture as their source of income and households encountered food shortages have the highest factor loadings, implying their highest contribution to increasing sensitivity of smallholder farmers and hence, mounting vulnerability of households to climate-related risks. In this study, although crop diversification has the largest factor loading (0.72, in Figure 2), implying its high contribution to reducing sensitivity of households, income diversification (off-farm income sources) of households was very low. Around 90% of households were solely dependent on agriculture as their income sources. As loss of crops and livestock because of climate-related risks can cause significant reductions of income which may force households to aid, large dependence on agriculture highly raises vulnerability of farmers. This is supported by findings of Seyoum (2015) who revealed that only 8% of farmers were participated in off-farm activities as their source of income and high dependency on climate sensitive livelihoods means that households are likely to be more vulnerable to climate change and variability.

On the other hand, higher factor loading of food shortage is explained by higher percentage of households who were exposed to food shortage. The survey results indicated that 77% households had encountered food shortage for time ranging from 2 to 3 months during normal seasons of the year suggesting their high sensitivity to climate-related shocks such as drought, flood, pests and diseases. The third indicator, households seen with sick family members have factor loadings of 0.48 and 0.45 for male- and female-headed households, respectively, which indicates their moderate contribution to sensitivity of households. The least contributing indicator to sensitivity of households is dependency ratio with factor loadings of 0.29 and 0.32 for men and women, respectively.

Furthermore, exposure of households to climate-related hazards is a crucial component of vulnerability of households. The survey results revealed that households were exposed for four climate-related hazards including drought, soil degradation, pests and diseases and flood. As indicated, drought has the largest factor loading (0.98) which implies that most households were exposed to drought amongst other climate-related risks (Figure 3). The results indicated that majority of households (81.8%) experienced drought four times in the last 10 years. Farmers complained that droughts have increased and recently, the known drought tolerant food crop, enset (*Enset ventricosum (Welw.) Cheesman*) has been drying in the study area. The second largest component of exposure was soil degradation with factor loadings of 0.54 and 0.58 for men and women’s farmlands exposure, respectively (Figure 3). More than half of the households (58%) reported that their farmlands were heavily exposed to soil degradation because of the occurrence of heavy rains. This was aggravated by ploughing of hilly areas because of the shortage of land as reported by key informants. Furthermore, the factor loadings of crop pests and diseases (0.5 and 0.45 for women and men’s crops, respectively) suggest that crops of households were also moderately exposed to pests and diseases associated with climate variability. Around 42% households complained that their crops especially cash crops were exposed to pests and diseases at least once in the last 5 years. Flooding has the least factor loading (0.2), suggesting that a small number of households were exposed to flood hazard in this study area. The survey results indicated that only 30% of households were exposed to flooding 2–3 times over the last 10 years.

### Vulnerability status of households

**FIGURE 4:**
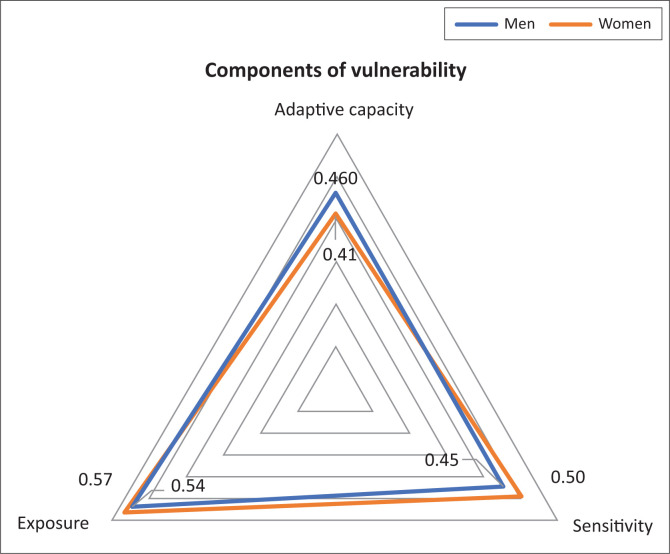
Triangle diagram showing the three contributing factors for vulnerability of households.

The three dimensions of vulnerability with their loadings which were used to develop vulnerability indices of households following the IPCC (2012) equation were presented (Figure 4). The results revealed the largest overall value of exposure for male- and female-headed households. As indicated (Figure 4), the diagram is obviously inclined towards exposure, implying that households were more exposed to climate-induced shocks than their capacity.

Therefore, exposure, with loadings of 0.54 and 0.57 for men and women, respectively, was the component which contributes the most to the vulnerability of households in the study area.

Furthermore, sensitivity towards climate-related hazards has an overall value of 0.45 and 0.5 for men and women, respectively, implying moderate sensitivity of the community in the study area. However, women in the community were more sensitive than their counterparts. The adaptive capacity, with a moderate overall value of 0.46 and 0.41 for men and women, respectively, implies that majority of the households were not capable of coping with adverse impacts of climate-induced shocks. The diagram clearly shows that the adaptive capacity of female-headed households was less than their counterparts because of their low access to public services and assets such as low access to climate information, credit, health, education, farmlands and low livestock assets.

**FIGURE 5:**
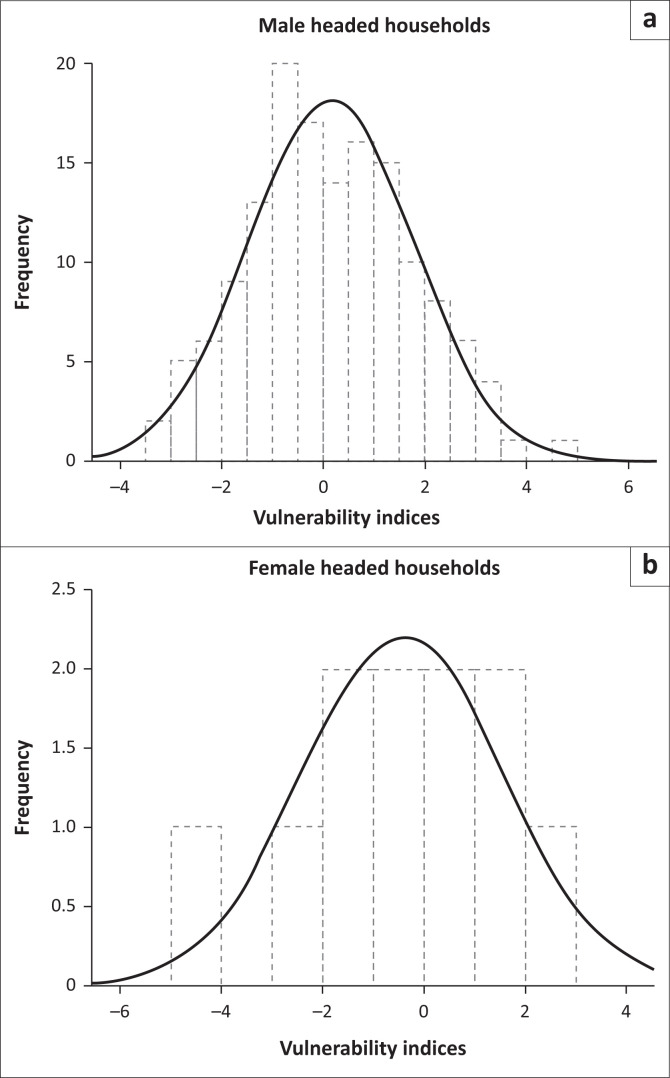
(a, b) Frequency distribution of male- and female-headed household’s vulnerability index.

Accordingly, four vulnerability statuses of male- and female-headed households were identified based on the graphed frequency of vulnerability indices of households, starting at 0 and ending in +6 and −6, increasing and decreasing by a factor of +2 and −2, respectively (Figures 5a and b). The chi-square test showed that there was a significant association between vulnerability situation and gender (Table 5). Thus, small groups of households, 12.8% of men and 9% of women were not vulnerable in the present situation whilst 37.7% and 27.3% of male- and female-headed households, respectively, were less vulnerable which means that they were in a vulnerable situation, but still capable to cope with climate-related shocks (Table 6).

**TABLE 5:** Chi-square tests to understand association between vulnerability status and gender.

Test statistics	Value	*df*	Sig.
Pearson chi-square	14.256†	3	0.003
Likelihood ratio	10.035	3	0.018
Linear-by-linear association	8.888	1	0.003
Number of valid cases	258	-	-

*df*, degrees of freedom; Sig. significance.

† , Four cells (15%) have expected count less than 5. The minimum expected count is 1.25.

**TABLE 6:** Vulnerability status of male- and female-headed households.

Vulnerability status	Vulnerability indices	Households (%)	
		Male-headed HHs	Female-headed HHs
Highly vulnerable	−2 to −6	8.5	18.2
Moderately vulnerable	−2.01 to 0	41.0	45.5
Less vulnerable	0.1 to 2	37.7	27.3
Non-vulnerable	+2.01 to +6	12.8	9.0

**Total**	**-**	**100**	**100**

HHs, household heads.

Results indicated that 41.0% and 45.5% male- and female-headed households, respectively, were moderately vulnerable to climate-related shocks, implying that those groups of households require temporary aid to recover during the occurrence of severe climate shocks. On the other hand, a small group of male-headed households (8.5 %) and relatively, somewhat a larger group of females-headed households (18.2%) were highly vulnerable to climate-related risks, implying that those groups of households are the most sensitive even for less intense climate shocks and require intensive care.

The overall average vulnerability indices of male- and female-headed households were −0.5 and −0.68, respectively, which imply that female-headed households were more vulnerable than male-headed households. This can be explained by high exposure and sensitivity of households and low level of adaptive capacity to cope with climate-induced risks. The present results are supported by the findings of Admassie, Adenew & Tadege (2008) in their study on climate change and adaptation strategies in Ethiopia who reported that female-headed households in Ethiopia were particularly vulnerable to climate change because of greater constraints to adaptation than male-headed households. Similar results were reported by Ongoro and Ogara (2012) who did their studies on the vulnerability of Samburu households in Kenya and revealed that female-headed households were more vulnerable to the impacts of climate variability than their men counterparts.

## Conclusion and recommendation

In this study area, the most challenging climate-related shocks and stress perceived by the local people were drought, soil degradation, pests and diseases and flood. The findings indicated that drought is found to be the most challenging climate-induced shock and a great concern of local people adversely affecting their agricultural productivity and production. Exposure of farmer’s farmlands to climate-induced soil degradation, exposure of crops to pests and diseases were also found to be high in the study area. Farmer’s high dependency on agriculture or low participation on off-farm activities, frequent occurrence of food shortage and diseases during normal season of the year and highly dependent ratios in households especially in female-headed households were found to be the most important factors that increase the sensitivity of households to climate-induced shocks. On the other hand, the adaptive capacity of households was not large enough to cope with climate shocks because of poor access of households to credit, market linkage and centre in the nearby villages, climate information, health services and shortage of farmlands. Consequently, a small group of male-headed households (8.5%) and relatively, somewhat a larger group of female-headed households (18.2%) were highly vulnerable whilst 41% and 45.5% of male- and female-headed households, respectively, were moderately vulnerable to climate-related shocks. The results confirmed that 37.7% and 27.3% of male- and female-headed households, respectively, were less vulnerable. The rest small groups of households, 12.8% of men and 9% of women were not vulnerable in the present situation, but they may come to vulnerable situation in the long-run if adaptive measures will not be taken by local decision makers to enhance their climate resilience.

Therefore, experts of the regional agriculture sector, researchers, administrative bodies and other concerned institutions and partners should mainstream climate change and variability in their development plan and develop an adaptation strategy to the increasingly becoming climate-related shocks. There is a need to enhance income diversification of farmers, soil and water conservation and afforestation of hilly areas. Farmers should be provided with drought and disease-tolerant crops and early maturing crops. Furthermore, large focus on women empowerment such as enhancing their access to affordable credit, market, climate information, health and access to and control over resources such as farmlands is paramount if farmers need to be climate resilient.
